# Retraction: Physio-metabolic and clinical consequences of wearing face masks—Systematic review with meta-analysis and comprehensive evaluation

**DOI:** 10.3389/fpubh.2023.1221666

**Published:** 2023-05-25

**Authors:** Frontiers Editorial Office

The journal retracts the 5 April 2023 article cited above.

Following publication, concerns were raised regarding the scientific validity of the article. An investigation was conducted in accordance with Frontiers' policies. It was found that the complaints were valid and that the article does not meet the standards of editorial and scientific soundness for Frontiers in Public Health; therefore, the article has been retracted.

This retraction was approved by the Chief Editors of Frontiers in Public Health and the Chief Executive Editor of Frontiers. The authors did not agree to this retraction.

